# Spontaneous Coronary Artery Dissection in the Late Third Trimester of Pregnancy

**DOI:** 10.7759/cureus.65968

**Published:** 2024-08-01

**Authors:** Nikolaos Antonakopoulos, Figalia Nika, Panagiota Tzela, Alexandros Tousis, Leonidas Antonakis, Periklis Davlouros, Georgios Adonakis

**Affiliations:** 1 Department of Obstetrics and Gynecology, School of Health Sciences, University of Patras, Patras, GRC; 2 Department of Midwifery, School of Health and Care Sciences, University of West Attica, Athens, GRC; 3 Department of Cardiology, School of Health Sciences, University of Patras, Patras, GRC

**Keywords:** pathophysiological changes in pregnancy, bilateral pleural effusions, tachypnea, acute-onset chest pain, percutaneous angiography, myocardial infarction, scad, spontaneous coronary artery dissection, pregnancy

## Abstract

Acute coronary syndrome due to a non-atherosclerotic, non-traumatic, or iatrogenic-induced spontaneous coronary artery dissection (SCAD) is a rare clinical condition that affects mostly young women of reproductive age. In this case, we present a 36-week-pregnant, 35-year-old G2P1 woman, with no previous medical history, who was admitted to our hospital with premature pre-labor contractions. During her hospitalization, she underwent a coronary artery percutaneous angiography revealing SCAD of the three coronary vessels, after an episode of acute-onset chest pain, tachypnea, EKG alterations, cardiac enzyme elevation, and bilateral pleural effusions. An emergency cesarean delivery was performed and the patient was transferred to the cardiology intensive care unit. Conservative management was decided and the woman was discharged a few days later.

## Introduction

Pregnancy is associated with a greater risk of thrombotic events compared to a non-pregnant state, acute coronary syndrome included. Spontaneous coronary artery dissection (SCAD), though extremely rare, is considered to be the major cause of antenatal myocardial infarction, leading to significant morbidity and mortality. The reported incidence is approximately two cases per 100,000 pregnancies. However, one out of four myocardial infarctions in pregnancy and 50% of post-partum coronary events are reportedly due to SCAD [1-3]. It usually affects women under 50 years with no cardiovascular risk factors [1,4,5]. It was first described in 1930 by Harold Pretty and the pathophysiological mechanism underneath this rare clinical condition still remains unclear and probably complex. Risk factors include hypertension, metabolic syndrome, ethnicity (African race), several forms of connective tissue disorders, and vasculitis, such as fibromuscular dysplasia, lupus erythematosus, and systemic inflammatory diseases. Especially for women, additional factors, such as multiparity, advanced maternal age, the postpartum period, prior use of oral contraceptive pills, infertility treatment, physical and emotional stress, as well as drug use, are mentioned in the literature [6,7].

The typical angiographic image of an intramural hematoma within the artery sets the diagnosis. This hematoma may get larger under arterial pressure and finally result in ischemia and infarction [8]. Clinical manifestations vary from a completely asymptomatic patient or minor atypical symptoms to cardiogenic shock or even sudden cardiac death. In the pregnant state, usually, it presents during the third trimester with a higher possibility of multi-vessel involvement.

Hemodynamic and vascular wall structure changes throughout pregnancy could be considered predisposing factors, but this is still a hypothesis. Increased total blood volume and cardiac output (hyperdynamic circulation), high concentration of sex hormones, specifically high levels of progesterone, alterations in smooth muscle cell proliferation and collagen synthesis, eosinophil lytic enzymes, changes in elastic fibers proportion compared to mucopolysaccharides content, and hypercoagulation state of pregnancy are some contributing factors that could lead to artery wall damage [9,10].

## Case presentation

A 35-year-old gravida 2 para 1 Caucasian woman was admitted to the Obstetric Emergency Department of the University Hospital of Patras due to premature pre-labor contractions at 35 weeks of gestation. She had an uncomplicated vaginal delivery two years ago, with free medical history in general. She was a non-smoker, had a BMI of 25, and no drug use was mentioned. Her risk for preeclampsia was low and she had regular antenatal care. A course of steroids for fetal pulmonary maturation was administered. On her 5th inpatient day, she presented a slight increase in C-reactive protein (CRP) as well as a borderline temperature of 37.4°C. Vaginal swab and urine cultures at admission came back negative. New cultures were sent and IV ceftriaxone was initiated. Fetal growth, Doppler, and biophysical profile were normal, but there were regular uterine contractions with no Bishop score changes.

**Figure 1:**
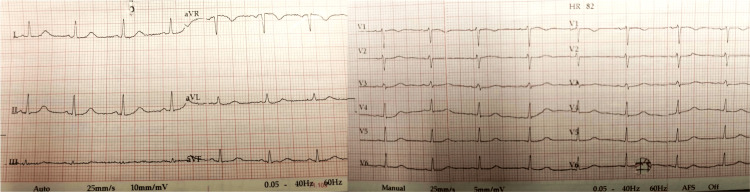
ECG showed sinus rhythm with no tachycardia and no ST abnormalities.

**Figure 2:**
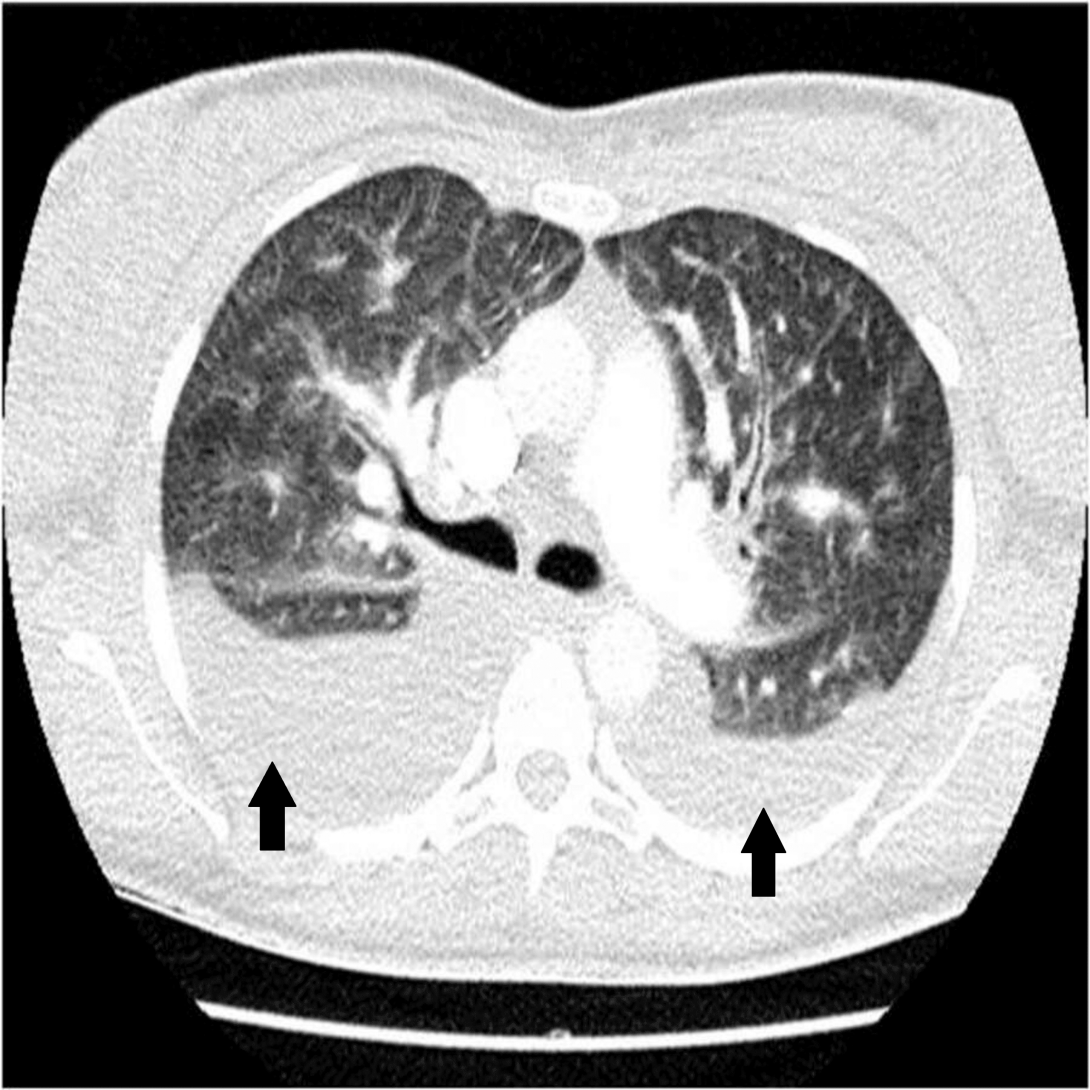
Computed tomography pulmonary angiography (axial view) showing significant pleural effusions (black arrows) bilaterally.

On the 6th inpatient day, she complained of sudden-onset chest pain, lasting several minutes, spreading to her back and upper limbs, along with mild dyspnea and nausea. She was hemodynamically stable with an oxygen saturation of 92%, with slight tachypnea. She also had a mild cough. Her clinical examination revealed no murmurs, normal heart sounds, no jugular distension, and no edemas, but there were pulmonary crackles bilaterally. Her electrocardiogram (ECG) revealed sinus rhythm with no tachycardia and no ST abnormalities (Figure 1). Her arterial blood gases showed signs of hypoxia with a base excess of -9.0 mmol/L. There were no signs of fetal distress. Her thyroid function was normal. A bedside heart echo was performed that showed a normal ejection fracture and no myocardial dysfunction. A computed tomography pulmonary angiography (CTPA) was also performed to exclude pulmonary embolism and other pulmonary morbidities, given that at this gestational age, the benefits outweigh the risks of radiation exposure. There were no signs of embolism, but significant pleural effusions bilaterally with peribronchial cuffing and a minor pericardial effusion were found (Figure 2). A few hours later, the woman’s dyspnea and cough had worsened and her systolic blood pressure was elevated at 150 mmHg to 160 mmHg. Serum troponin was elevated (1771 pg/ml) and a new heart echo revealed a minor pericardial effusion, mild left ventricular dysfunction with ejection fraction of 50-55%, hypokinetic lower and posterior wall, and mild mitral valve regurgitation. Diuretics were administered and the patient’s clinical condition improved. After a multidisciplinary discussion, a cesarean section under general anesthesia was decided and a healthy baby girl of 2790 g was delivered, at 36 weeks of gestation, with good Apgar scores of 8/1, 9/5, and 10/10.

**Figure 3:**
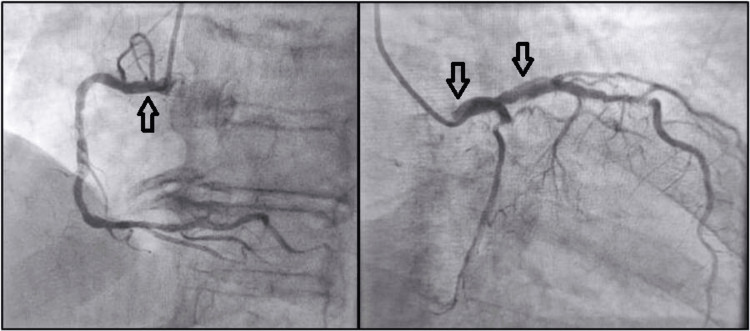
Coronary artery angiography confirmed the diagnosis of spontaneous coronary artery dissection. Arrows indicate the site of arterial wall dissection.

The woman was transferred to the cardiology ICU, where oxygen therapy, beta-blockers, diuretics (furosemide), and anti-hypertensive medication were administered, given that her clinical picture was compatible with acute heart failure (highly sensitive troponin peak of 2368 pg/ml, B-type natriuretic peptide (BNP) of 950 pg/mL). The woman underwent a coronary artery angiography and the spontaneous coronary artery dissection diagnosis was set with all three coronary branches being involved (right coronary artery dissection, as well as in the middle of the anterior descending branch, with an extension of the dissection into the left circumflex artery) (Figure 3). Anti-platelets and anti-coagulants were initiated. Conservative treatment was decided and she was discharged a few days later. Her clinical condition was stable and close follow-up with a new coronary angiography after one month was recommended. The patient was also advised to avoid intense physical activity and emotional stress.

## Discussion

Antenatal acute cardiac events are rare but potentially life-threatening. Chest pain in a pregnant woman always requires further investigation. Differential diagnosis should include pericarditis or myocarditis, pneumonia, pulmonary embolism, aortic dissection, peripartum cardiomyopathy, SCAD, myocardial infarction, spontaneous pneumothorax, or even gastrointestinal disorders, such as gall bladder disease and gastroesophageal reflux.

SCAD is an ischemic event that occurs with an expanding intramural hematoma between the tunica media and intima of an artery resulting in separation of the intima‐media complex. This creates a false lumen, which in turn compresses the true lumen of the vessel resulting in ischemia and therefore an acute coronary syndrome [8]. Although almost 40% of myocardial infarction incidences that take place during pregnancy and postpartum are due to SCAD, this condition still remains underdiagnosed in many cases. What is also true is that most of the cases present at the peripartum and postpartum period and less than one in five cases present antenatally [10].

Hemodynamic and hormonal changes during pregnancy can be contributing factors for SCAD. For those presenting with atypical symptoms, investigation typically starts with serial troponin levels in addition to serial ECG and heart echo. The final diagnosis is only made via invasive coronary angiography for which there is significant concern surrounding fetal radiation, as there is a strong association between procedural exposure and childhood cancer [11]. In our case, we proceeded to deliver the fetus so as to be able to perform diagnostic procedures and treatment without fetal concerns. The vast majority of pregnancy-associated SCAD cases occur in the postpartum period; therefore, data on delivery planning in the context of antenatal SCAD are very limited.

Mortality rates of pregnancy-related SCAD with myocardial infarction are high [12,13]. Percutaneous intervention (PCI) is not routinely performed in cases of SCAD in comparison with atherosclerotic infarctions where this is the treatment of choice. The main reason why SCAD is preferably treated conservatively is due to the risk of expanding the hematoma during the coronary artery intervention technique [14]. This iatrogenic complication could result in severe ischemia possibly fatal for the patient. When PCI or coronary artery bypass graft is indicated, they are performed after delivering the baby [14,15]. Features that must be taken into consideration before any decisions are made include the number of vessels that are involved in SCAD, the exact site of arterial wall dissection, the hemodynamic profile of the patient, and the availability of medical services required to perform the indicated operation [10,15,16].

## Conclusions

Antenatal acute cardiac events are rare but potentially life-threatening. SCAD, though extremely rare, is considered to be the major cause of antenatal myocardial infarction, leading to significant morbidity and mortality. It is of great importance for the pregnant woman to be assessed by a multidisciplinary team. The gold standard of diagnosis is coronary artery angiography. Conservative management is preferred when possible, as the risk of iatrogenic complications during coronary artery intervention could be catastrophic. When PCI or coronary artery bypass graft is indicated, they are performed after delivering the baby. Our case highlights that maternity care professionals should always address any signs or symptoms that suggest a rare cardiac event during gestation with caution and a thorough medical history and physical examination should follow, bearing in mind that both maternal and fetal health are threatened.
